# High performance anode-supported tubular solid oxide fuel cells fabricated by a novel slurry-casting method

**DOI:** 10.1038/srep08174

**Published:** 2015-02-02

**Authors:** Nan-Qi Duan, Dong Yan, Bo Chi, Jian Pu, Li Jian

**Affiliations:** 1 Center for Fuel Cell Innovation, State Key Laboratory of Coal Combustion, School of Materials Science and Engineering, Huazhong University of Science and Technology, Wuhan, Hubei 430074, China

## Abstract

Tubular solid oxide fuel cells were fabricated and evaluated for their microstructure and electrochemical performance. The tubular substrate was prepared by casting NiO-Y_2_O_3_ stabilized ZrO_2_ (YSZ) slurry on the inner wall of a plastic mold (tube). The wall thickness and uniformity were controlled by slurry viscosity and rotation speed of the tube. The cells consisted of Ni-YSZ functional anode, YSZ electrolyte and (La_0.8_Sr_0.2_)_0.95_MnO_3-δ_ (LSM)-YSZ cathode prepared in sequence on the substrate by dip-coating and sintering. Their dimension was 50 mm in length, 0.8 mm in thickness and 10.5 mm in outside diameter. The peak power density of the cell at temperatures between 650 and 850°C was in the range from 85 to 522 mW cm^−2^ and was greatly enhanced to the range from 308 to 1220 mW cm^−2^ by impregnating PdO into LSM-YSZ cathode. During a cell testing at 0.7 A cm^−2^ and 750°C for 282 h, the impregnated PdO particles grew by coalescence, which increased the cathode polarization resistance and so that decreased the cell performance. According to the degradation tendency, the cell performance will be stabilized in a longer run.

A solid oxide fuel cell (SOFC), consisting of porous anode and cathode separated by a dense electrolyte, is an electrochemical device, which efficiently and environment friendly converts the chemical energy of fossil and hydrocarbon fuels into electricity and heat without involving combustion and mechanical motion. The operating temperature of SOFCs was near 1000°C, and has been lowered to the intermediate-temperature range between 650 and 800°C for the benefits in materials selection, performance durability and manufacturing cost by taking the anode-supported and thin electrolyte cell configuration. It is also realized that the electrocatalytic activity of conventional cathode materials, such as Sr-doped LaMnO_3_, is lower at intermediate temperatures and needs to be improved for achieving high cell performance.

The most popular types of cell design for SOFCs are tubular and planar. The tubular cell has advantages in sealing, cell-to-cell connection^1^, thermal cycling and start-up because of its symmetric geometry^2^. Many techniques for tubular cells fabrication have been reported in recent years, such as extrusion^3^,^4^,^5^,^6^,^7^, iso-pressing^8^ slip casting^9^ and dip-coating^10^,^11^. In the present study, a novel “slurry-casting” method was developed for preparing the tubular anode-support, on which functional anode and electrolyte were dip-coated in sequence before co-firing. This method is easy to operate, cost effective and applicable to both laboratory and industry scale fabrication of tubular cells. In this paper, the microstructure and performance of such prepared tubular cells are reported. The cells consisted of conventional Ni-Y_2_O_3_ stabilized ZrO_2_ (Ni-YSZ) cermet anode, YSZ electrolyte and (La_0.8_Sr_0.2_)_0.95_MnO_3-δ_ (LSM)-YSZ cathode; and their performance was greatly enhanced by Pd modification of the cathode.

## Results

**Figure 1:**
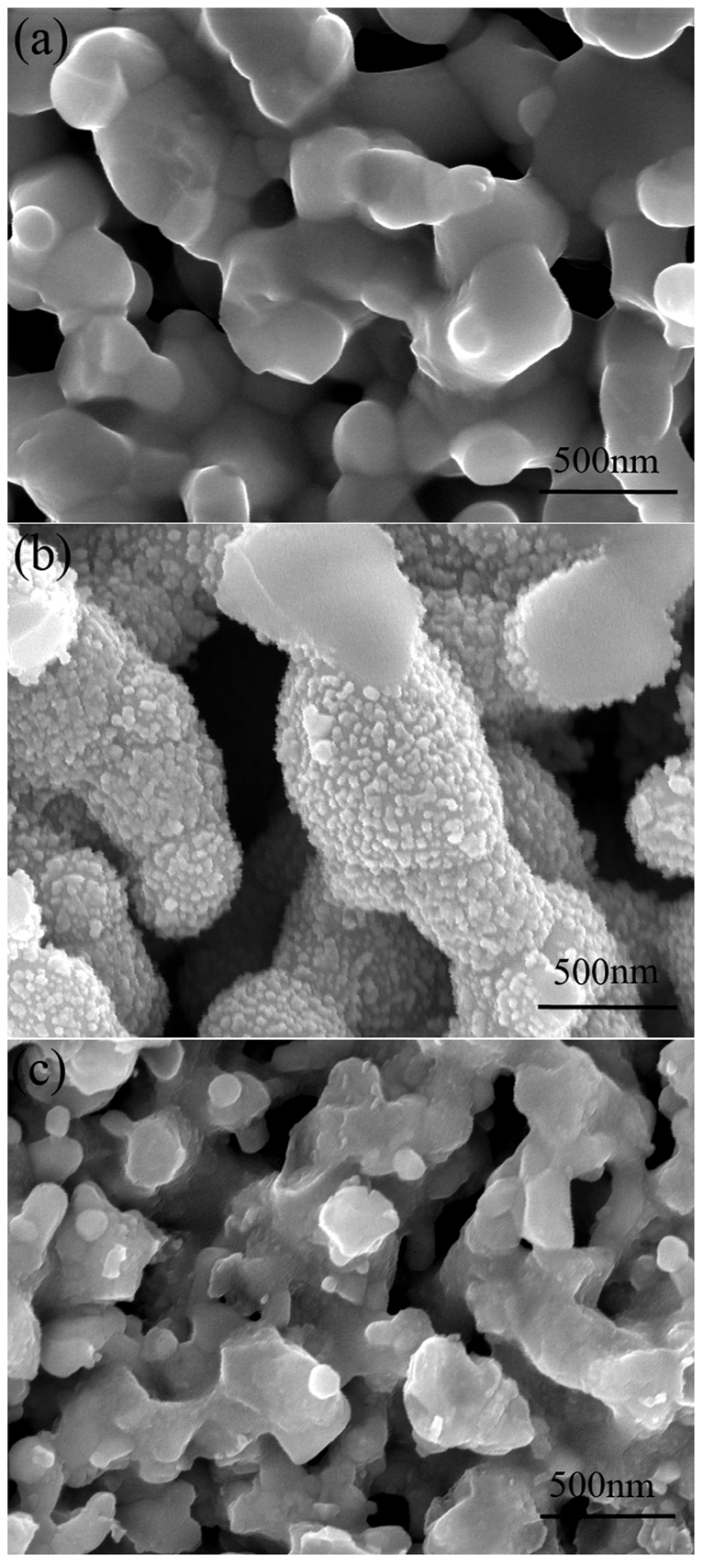
The cross-sectional microstructure of cathodes: (a) LSM-YSZ, (b) as-prepared Pd+LSM−YSZ and (c) tested Pd+LSM−YSZ at 0.7A cm^−2^ and 750°C for 132 h.

The sintered cell was 50 mm in length, ~0.8 mm in wall thickness and ~10.5 mm in outside diameter (Supplementary Fig. S1). After reduction during the cell test, the porosity of the anode-support was about 38%; and the functional anode, electrolyte and cathode were well adhered to each other with a uniform thickness of approximately 25, 15 and 15 μm (Supplementary Fig. S2), respectively. In the Pd-modified LSM-YSZ cathode (Pd+LSM−YSZ), nano-sized PdO particles (20–50 nm) were uniformly distributed on the LSM-YSZ scaffold by solution impregnation. Fig. 1 demonstrates the microstructure of the as-prepared LSM-YSZ (Fig. 1a) and Pd+LSM−YSZ (Fig. 1b) cathodes and the tested Pd+LSM−YSZ (Fig. 1c) cathode.

**Figure 2:**
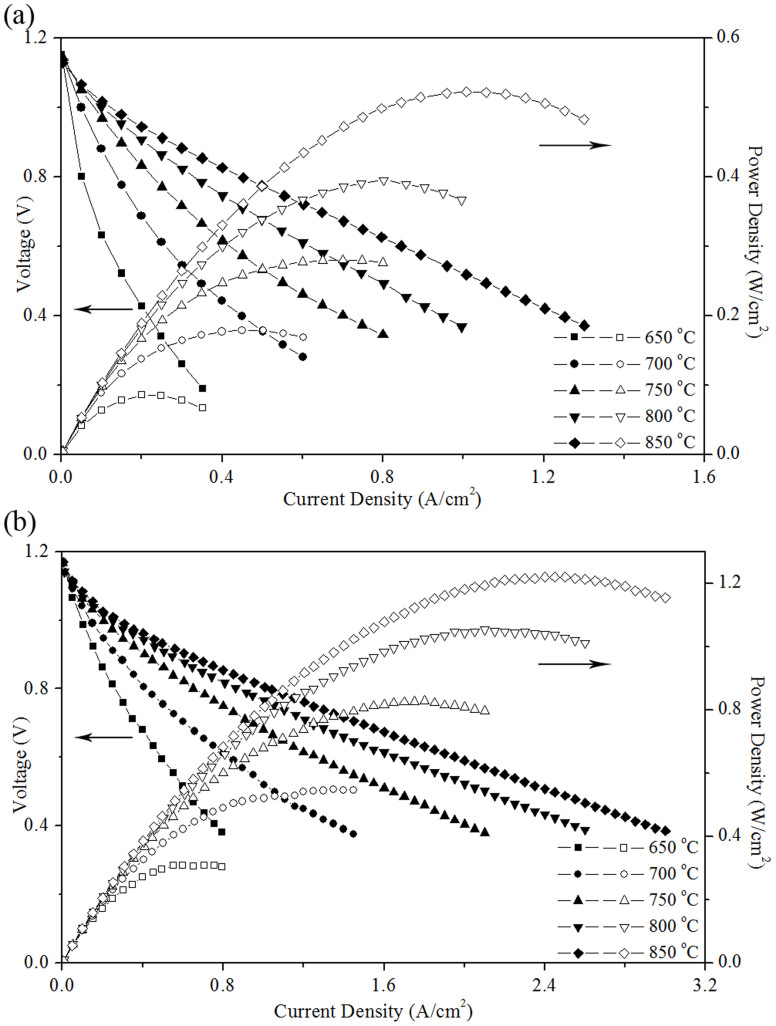
The performance of the anode-supported tubular cells without (a) and with (b) impregnated PdO particles in the cathode at various temperatures.

Fig. 2 shows the I-V-P curves of prepared cells (active area 3 cm^2^) at temperatures between 650 and 850°C with H_2_ as the fuel and air as the oxidant. Their open circuit voltage was higher than 1.1 V, suggesting that the cells were properly sealed and the electrolyte was gas tight without fuel crossover. Without adding PdO particles into LSM-YSZ cathode, the peak power density varied from 85 to 522 mW cm^−2^ (Fig. 2a) at temperatures from 650 to 850°C; whereas it was increased more than twice to the range from 308 to 1220 mW cm^−2^ (Fig. 2b) at the same temperatures by impregnating PdO particles into the cathode.

**Figure 3:**
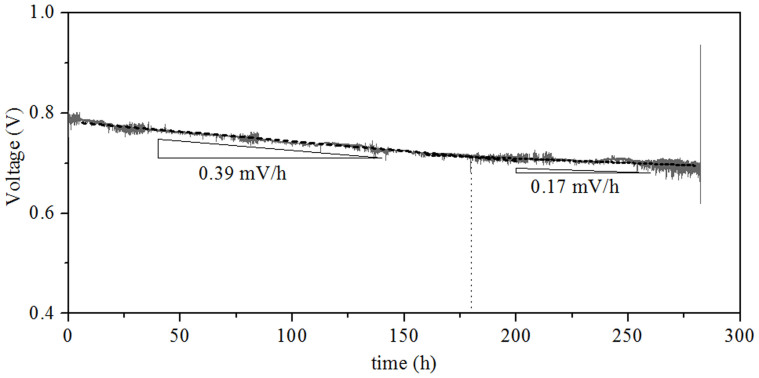
Long-term performance of the anode-supported tubular cell with Pd+LSM−YSZ cathode at 0.7 A cm^−2^ and 750°C.

In order to evaluate the long-term performance of the cell with Pd+LSM-YSZ cathode, it was tested at 0.7 A cm^−2^ and 750°C for 282 h, as shown in Fig. 3. After the initial voltage increasing from 0.753 to 0.782 V, possibly due to electrochemical activation of the LSM-YSZ^12^,^13^, the voltage decreased almost linearly at a rate of 0.39 mV h^−1^ for around 170 h before the degradation rate changed to a lower value of 0.17 mV h^−1^.

## Discussion

As shown in Fig. 2, the cell performance at open circuit voltage and temperatures between 650 and 850°C was greatly enhanced by impregnating PdO particles into the LSM-YSZ cathode. This phenomenon was intensively studied^14^,^15^,^16^ in our group. It was found that cathode polarization resistance was greatly reduced, especially for the low-frequency one, due to the redox reaction between Pd and PdO that facilitated the low-frequency electrode processes of oxygen adsorption and dissociation in the cathode.

**Figure 4:**
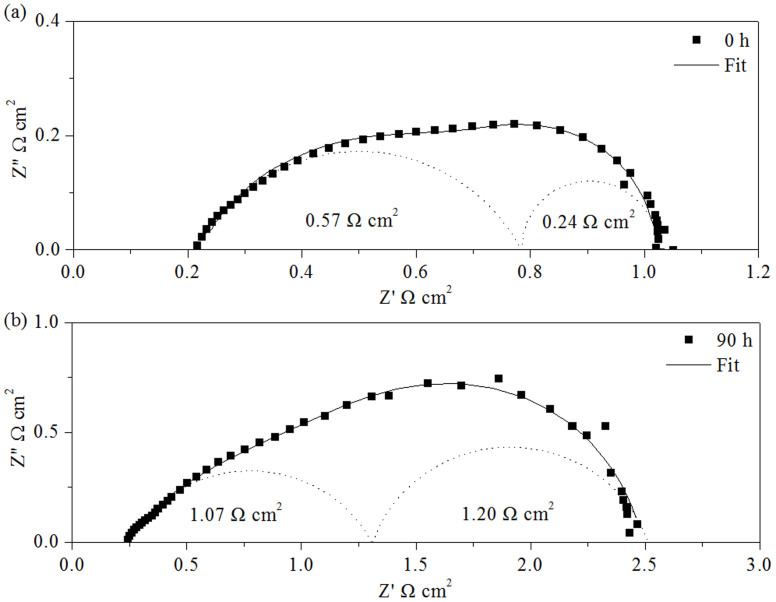
Electrochemical impedance spectra of the anode-supported cells with Pd+LSM−YSZ cathode measured at (a) 0 and (b) 90 h during the performance test at 750°C under a current density of 0.7 A cm^−2^.

It was also observed that the performance of the cell with Pd+LSM−YSZ cathode decreased at a gradually slowed rate over time at 0.7 A cm^−2^ and 750°C (Fig. 3). Since the Ni-YSZ anode, electrolyte and LSM-YSZ scaffold are stable under the testing conditions^17^,^18^, as demonstrated by Supplementary Fig. S3 for the durability of the cell with the LSM-YSZ cathode, the performance degradation is expected to be related to the microstructure change of the impregnated particles^19^,^20^. The original nano-sized PdO particles coalesced into larger particles on the surface of the scaffold during the test at 750°C, as shown in Fig. 1c, which decreased the surface area of the impregnated particles and triple phase boundary, and in turn the cell performance. Fig. 4 shows the electrochemical impedance spectra of the cell measured at 0 and 90 h during the durability test. The ohmic resistance remained almost constant at around 0.22 Ω cm^−2^, but the polarization resistance increased from 0.81 to 2.27 Ω cm^−2^. The electrochemical performance of the Ni-YSZ anode and LSM-YSZ cathode was proved to be stable (Fig. S3); therefore it is reasonable to consider that the polarization resistance increase was related to the infiltrated PdO particles. By data fitting the impedance spectra according to the equivalent circuit shown in Fig. 4, the high- and low-frequency polarization resistances were determined. The low-frequency polarization resistance was increased by almost 5 times from 0.24 to 1.20 Ω cm^2^ and the high-frequency one was increased by less than 2 times from 0.57 to 1.07 Ω cm^2^. This result provides another evidence for that the cell performance was compromised by the increase of both the low- and high-frequency polarization resistances, particularly the low-frequency one, due to the growth of the impregnated particles. As the growth rate of the impregnated particles slows down, the cell performance will gradually approach to a stable level (Fig. 3). To avoid such cell performance degradation, the microstructure of the Pd+LSM−YSZ cathode should be stabilized by adding alloying elements into PdO particles^20^.

## Methods

### Fuel cell fabrication

The tubular substrate with one closed end was made from the composite powder consisting of 57 wt% NiO and 43 wt% YSZ by a slurry-casting method. The slurry was prepared by ball milling for 24 h with xylene and ethanol as the solvents, fish oil as the dispersant, corn starch as the pore former, polyvinyl butyral as the binder and butyl benzyl phthalate as the plasticizer. The ball-milled homogeneous slurry was poured into a tubular plastic mold and degassed, while it was rotated at a speed of 2000 r min^−1^ for 2 min in a centrifugal machine. Slurry viscosity was carefully adjusted to between 16000 and 20000 mPa s, so that it wetted the plastic mold perfectly, forming a layer of slurry that covered the inner wall of the mold after extra slurry was poured out in a container for reuse. The slurry hanging on the wall of the mold was gradually dried and detached from the mold automatically due to shrinkage, while the mold was rotated vertically on a rotating plate at a constant angular velocity. The thickness and uniformity of the substrate tube was controlled by slurry viscosity and rotating speed. Finally, a crack-free green tubular NiO-YSZ substrate was obtained with a uniform wall thickness and smooth surfaces (Supplementary Fig. S1). The dried substrate was heated slowly for debinding and pre-sintered at 1000°C. For fabrication of the functional anode and electrolyte, slurries containing 30 wt% NiO-YSZ (same composition as above) and 40 wt% YSZ, respectively, were prepared with terpilenol or ethyl alcohol as the solvent and ethyecellulose or polyvinyl butyral as the binder, and dip-coated in sequence on the outer surface of the pre-sintered NiO-YSZ substrate (Supplementary Fig. S1), prior to co-firing at 1390°C for 3.5 h. Composite LSM-YSZ cathode, containing 50 wt% LSM and 50 wt% YSZ, was applied on the top of the YSZ electrolyte also by dip-coating using a slurry prepared in a similar way to the above, and fired at 1150°C for 2 h to obtain a tubular anode-supported cell. To enhance the cell performance, PdO was introduced into the LSM-YSZ composite cathode by impregnation of PdCl_2_ solution with a Pd concentration of about 0.5 mol L^−1^ and subsequent calcination at 700°C in air for 1 h. Ammonium hydroxide was added into the solution to adjust pH value to 8. The loading of the impregnated PdO in the cathode was about 1.2 mg cm^−2^ after repeating the process for 5 times.

### Fuel cell test

The cell performance was evaluated by using an in-house developed testing setup (Supplementary Fig. S4). The cell was sealed by using a ceramic sealant. Ni foam was rolled up and squeezed into the tubular cell as the anode current collector. Pt paste painted on the cathode and Ag wire were used as the cathode current collector. Pure H_2_ was fed to the anode at a rate of 200 ml min^−1^; and ambient air was blown into the furnace at a rate of 300 ml min^−1^.

### Characterizations

Electrochemical measurement was carried out by using a Solartron 1260 frequency response analyzer (Solartron Analytical Ltd.) in a frequency range from 1000 kHz to 0.1 Hz with a signal amplitude of 10 mV at open circuit. The long term durability test was performed by using a DC power source (IT-6720, iTech). The microstructure of the cell was examined by a scanning electron microscope (SEM, Sirion 200 and Quanta 200, Holland FEI Company).

## Author Contributions

N.-Q.D. and D.Y. conducted the experiments and prepared the manuscript; B.C. and J.L. provided suggestions to the experiments; P.J. discussed the results and revised the manuscript.

## Supplementary Material

Supplementary Information Supporting Online Materials
