# Supplementary material for: High performance anode-supported tubular solid oxide fuel cells fabricated by a novel slurry-casting method
Source: Sci Rep. 2015 Feb 2;5:8174. doi: 10.1038/srep08174 (PMC4313086; doi:10.1038/srep08174)
Supplement: Supplementary Information — Supporting Online Materials [file srep08174-s1.pdf]

Supporting Online Materials

High performance anode-supported tubular solid oxide fuel cells fabricated by a novel slurry-casting method

Nan-Qi Duan, Dong Yan, Bo Chi, Jian Pu*, Li Jian

Center for Fuel Cell Innovation, State Key Laboratory of Coal Combustion, School of Materials Science and Engineering, Huazhong University of Science and Technology, Wuhan, Hubei 430074, China

*Corresponding author. Tel.and fax: +86 27 87558142.

E-mail address: pujian@hust.edu.cn (J. Pu).

Characterization

Fig. S1 shows the pictures of green tubular substrate, dipped and sintered tubular cells. The sintered cell was shrunk 20% in length and 12% in outside diameter to a final dimension of 50 mm (in length) × 0.8 mm (thickness) × 10.5 mm (outside diameter).

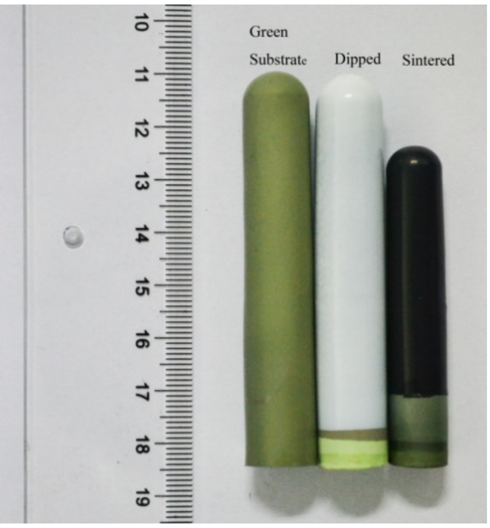

Figure S1| The green tubular substrate, dipped and sintered tubular cells.

Fig. S2 shows the cross-sectional microstructure of the anode-supported cell tested at temperatures between 650 and 850 °C. After reduction during the test, the anode-support was about 38% porous; and the functional anode, electrolyte and cathode well adhered to each other with a uniform thickness of approximately 25, 15 and 15 μm, respectively.

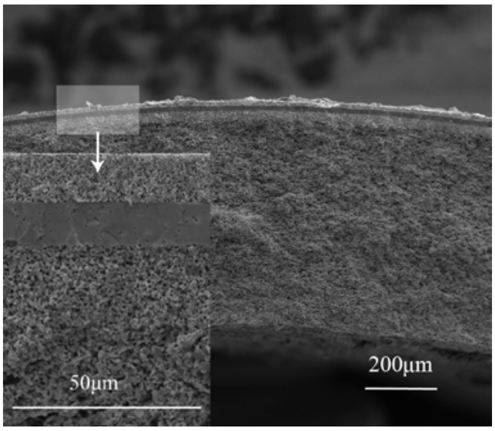

Figure S2| The cross-sectional microstructure of the anode-supported cell tested at temperatures between 650 and 850 °C.

Fig. S3 shows the time dependence of voltage for a tubular cell with LSM-YSZ cathode at 0.25 A cm^{-2} and 750 °C. The voltage increased very fast from initial value of 0.746 to 0.860 V within first 24 h and then slowly in the rest time of the test. Within the total testing time of 120 h, the cell showed no voltage degradation, demonstrating the stability of LSM-YSZ cathode and Ni-YSZ anode under the testing conditions.

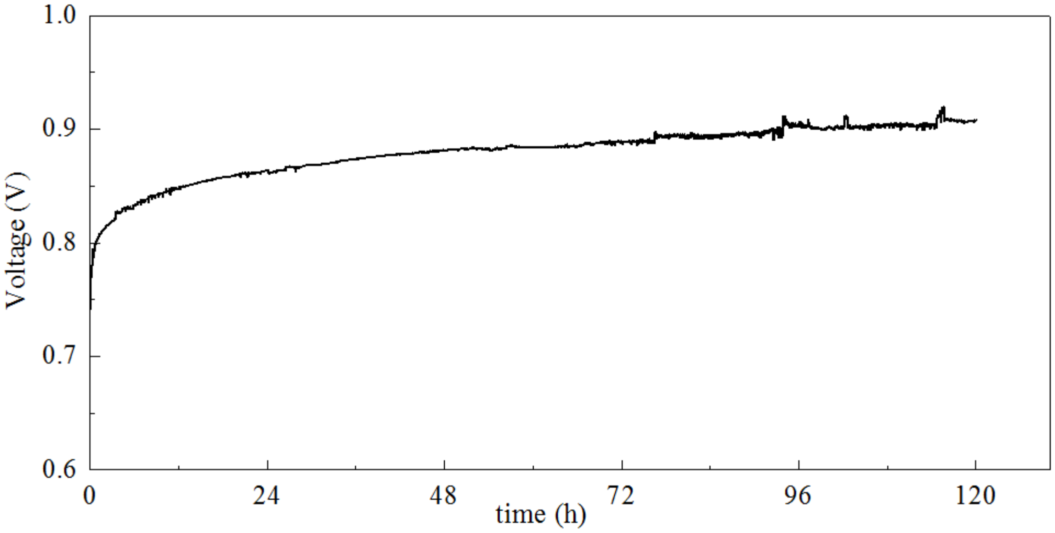

Figure S3| Voltage of the cell with LSM-YSZ cathode as a function of testing time at 0.25 A cm^{-2} and 750 °C.

The cell performance was evaluated by using an in-house developed testing setup. Fig. S4 shows the schematic diagram of the testing setup. Ni foam was rolled up and squeezed into the tubular cell as the anode current collector. Silver wores was used to current leading wires.

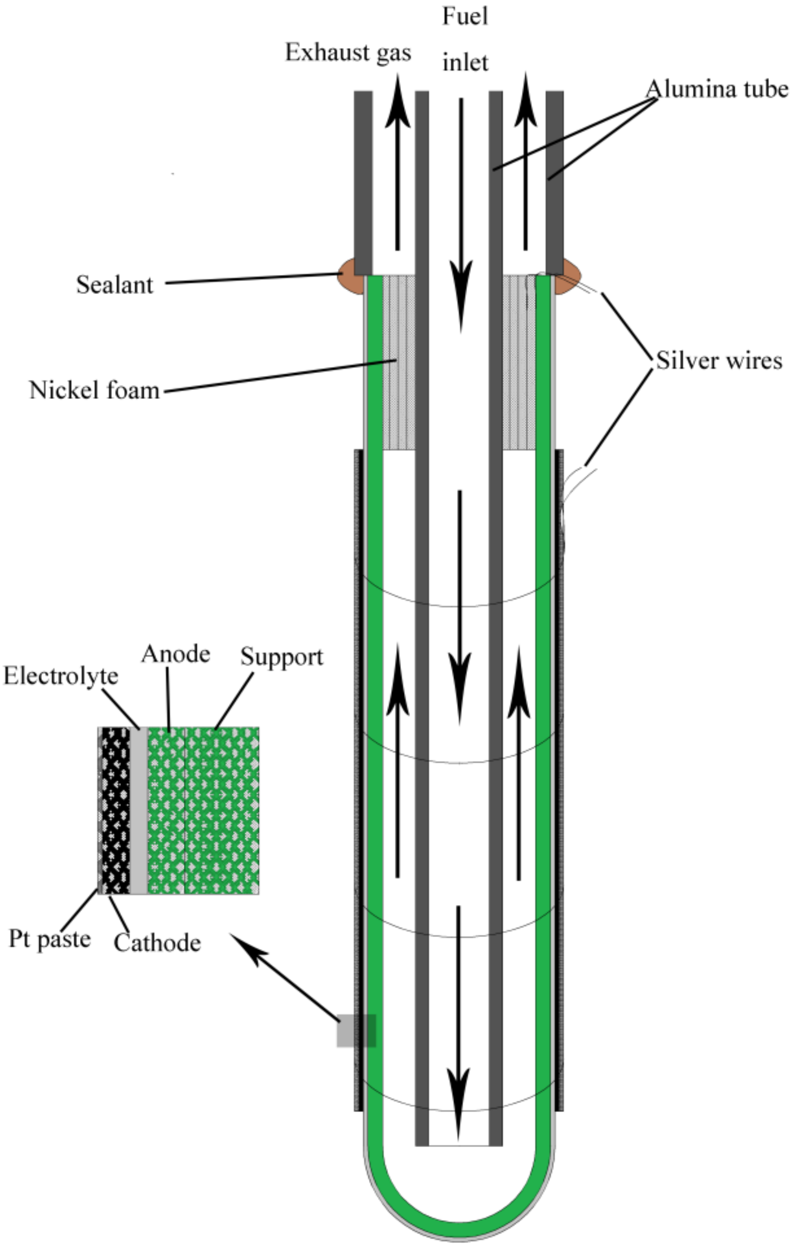

Figure S4| Schematic diagram of the testing setup for performance evaluation of the anode-supported tubular cell.
